# GNUV201, a novel human/mouse cross-reactive and low pH-selective anti-PD-1 monoclonal antibody for cancer immunotherapy

**DOI:** 10.1186/s12865-024-00609-z

**Published:** 2024-05-11

**Authors:** Hae-Mi Kim, Kyoung-Jin Kim, Kwanghyun Lee, Myeong Jin Yoon, Jenny Choih, Tae-Joon Hong, Eun Ji Cho, Hak-Jun Jung, Jayoung Kim, Ji Soo Park, Hye Young Na, Yong-Seok Heo, Chae Gyu Park, Heungrok Park, Sungho Han, Donggoo Bae

**Affiliations:** 1 Genuv Inc., B1 Shinyoung Building, 14 Gyeonghuigung-gil, Jongno-gu, Seoul, Republic of Korea; 2 Genuv US Subsidiary, CIC, 1 Broadway, Cambridge, MA USA; 3 Laboratory of Immunology, Severance Biomedical Science Institute, Yonsei University College of Medicine, Seoul, Republic of Korea; 4 Brain Korea 21 PLUS/FOUR Project for Medical Science, Yonsei University College of Medicine, Seoul, Republic of Korea; 5 Department of Neurology, Severance Hospital, Yonsei University College of Medicine, Seoul, Republic of Korea; 6 Department of Chemistry, Konkuk University, 120 Neungdong-Ro, Gwangjin-Gu, Seoul, 05029 Republic of Korea

**Keywords:** Anti-PD-1 antibody, Monoclonal antibody, Cross-reactive, TME selective, Cancer immunotherapy

## Abstract

**Background:**

Several PD-1 antibodies approved as anti-cancer therapies work by blocking the interaction of PD-1 with its ligand PD-L1, thus restoring anti-cancer T cell activities. These PD-1 antibodies lack inter-species cross-reactivity, necessitating surrogate antibodies for preclinical studies, which may limit the predictability and translatability of the studies.

**Results:**

To overcome this limitation, we have developed an inter-species cross-reactive PD-1 antibody, GNUV201, by utilizing an enhanced diversity mouse platform (SHINE MOUSE™). GNUV201 equally binds to human PD-1 and mouse PD-1, equally inhibits the binding of human PD-1/PD-L1 and mouse PD-1/PD-L1, and effectively suppresses tumor growth in syngeneic mouse models. The epitope of GNUV201 mapped to the “FG loop” of hPD-1, distinct from those of Keytruda^®^ (“C’D loop”) and Opdivo^®^ (N-term). Notably, the structural feature where the protruding epitope loop fits into GNUV201’s binding pocket supports the enhanced binding affinity due to slower dissociation (8.7 times slower than Keytruda^®^). Furthermore, GNUV201 shows a stronger binding affinity at pH 6.0 (5.6 times strong than at pH 7.4), which mimics the hypoxic and acidic tumor microenvironment (TME). This phenomenon is not observed with marketed antibodies (Keytruda^®^, Opdivo^®^), implying that GNUV201 achieves more selective binding to and better occupancy on PD-1 in the TME.

**Conclusions:**

In summary, GNUV201 exhibited enhanced affinity for PD-1 with slow dissociation and preferential binding in TME-mimicking low pH. Human/monkey/mouse inter-species cross-reactivity of GNUV201 could enable more predictable and translatable efficacy and toxicity preclinical studies. These results suggest that GNUV201 could be an ideal antibody candidate for anti-cancer drug development.

**Supplementary Information:**

The online version contains supplementary material available at 10.1186/s12865-024-00609-z.

## Background

The quality of the immune system is based on a precise balance between activating and inhibiting signals to maintain immune homeostasis. Immune checkpoints act as inhibiting signals whose role is to prevent the immune system from overactivation that could harm normal cells in the body. Some cancer cells protect themselves from being eliminated by the immune system through exploiting immune checkpoints, making the cancers refractory to conventional anti-cancer therapies. Immunotherapy called immune checkpoint inhibitors that block inhibitory immune checkpoint signals on T cells can lead to reactivation of anti-tumor immune responses. Among the immune checkpoints, programmed cell death 1 (PD-1) is one of the best-characterized molecules [1, 2]. Signaling of PD-1 begins with its ligand, PD-L1, which is highly expressed on the surface of cancer cells. The interaction of PD-1 on T cells and PD-L1 on cancer cells allow cancer cells to escape T-cell immune responses. Therefore, inhibition of the binding of PD-1 and PD-L1 with antibodies restores T cell function and further prevents cancer cells from evading immune surveillance [3, 4]. The reason PD-1 is in the spotlight is because it has a relatively better balance between efficacy and toxicity than the other immune checkpoints such as CTLA-4, LAG-3, TIM-3, TIGIT, and BTLA, and its clinical efficacy and mechanism of action are well established [5, 6].

Anti-PD-1 antibodies, such as Keytruda^®^ and Opdivo^®^, have been approved for use in a wide variety of solid tumors [7–11]. and various new antibodies are undergoing clinical trials. Although anti-PD-1 therapy has shown impressive efficacy, clinical data of anti-PD-1s have shown a limited response rate. In most cancers, durable response only occurred in a small portion of patients, while a large group of patients suffered primary resistance and some of the responders developed acquired resistance [12]. Various combination strategies with anti-PD-1 antibodies have been tested in preclinical and clinical stages to overcome these limitations.

In general, developing new therapeutic antibodies requires efficacy and safety data from animal models to predict optimal dose range which would be both efficacious and safe for human trials [13, 14]. For most candidate antibodies that can only bind to the human target protein, obtaining various meaningful preclinical data that can precisely predict efficacy and safety in humans is limited [15]. To address this issue, a surrogate antibody functionally equivalent to the therapeutic antibody that binds specifically to the target ortholog expressed in the intended animal species or surrogate animal models such as transgenic mice carrying the human target are used. However, it is unclear how well the surrogate preclinical options can represent the exact human target pharmacology in terms of efficacy and side effects [16]. Therefore, an antibody equally cross-reactive to the target of both human and relevant preclinical models can be a potential solution to enhance the predictability of clinical outcomes and the success rates of clinical trials that are designed based on relevant preclinical pharmacodynamic and pharmacokinetic studies.

Inter-species cross-reactive anti-PD-1 is needed to de-risk the majority of combination clinical trials of anti-PD-1s with other immunotherapies, targeted therapies, chemotherapies, and radiotherapies to overcome the limitations of anti-PD-1 monotherapy [17, 18] since all of the approved anti-PD-1s, including Keytruda^®^ and Opdivo^®^, are not cross-reactive to mouse PD-1 (mPD-1). For better prediction of efficacy and safety of anti-PD-1 in clinical trials, anti-PD-1 that binds to both humans and mice is required, but the sequence homology of the extracellular domain (ECD) of hPD-1 and mPD-1 is low (61.2%), making it difficult to develop human/mouse cross-reactive antibodies. Therefore, we aimed to generate an antibody that cross-reacts with both hPD-1 and mPD-1 using a mouse platform to generate antibodies with enhanced diversity (SHINE MOUSE™) with selected immunization methods.

Anti-PD-1 s have a relatively favorable toxicity profile, but blocking of PD-1 immune checkpoint sometimes causes the development of serious toxicity through reactivation of the immune system and imbalance of immune tolerance, also known as immune-related adverse events [19, 20]. These events may affect multiple organ systems and tissues, with clinical symptoms of autoimmune-like or inflammatory adverse effects such as thyroid disorders, pneumonitis, and hepatitis, which are possibly due to the infiltration of activated immune cells promiscuously into multiple organs and tissues [21]. Therefore, we also aimed to develop anti-PD-1 that is highly selective for the tumor microenvironment (TME).

Our results suggest that GNUV201 could be a promising candidate for immune oncology therapy, not only as a monotherapy but also as an excellent partner molecule for combination therapies. Its mouse/human cross-reactivity would enhance the predictability and translatability of efficacy and safety from various pre-clinical models to clinical trials. Furthermore, GNUV201 can be utilized as a T cell-specific targeting arm for various multi-specific biologics, based on its specific binding to PD-1 that is highly expressed on exhausted T cells. In addition, higher affinity of GNUV201 for PD-1 at TME-mimicking low pH provides additional selectivity, which could reduce adverse effects and maximize efficacy [22].

## Methods

### Animal

SHINE MOUSE™ in the C57BL/6 background (Macrogen. Inc.) were maintained in specific pathogen-free facilities during the period of immunization with antigens. Animal care and experiments were carried out in accordance with the guidelines set by the institutional animal care and use committee (IACUC) of Yonsei University College of Medicine.

7-week-old female C57BL/6 mice were purchased from Orient Bio (Seongnam, Republic of Korea). Mice were maintained in a specific pathogen‐free facility at the animal center of Genuv Inc. in accordance with the Guide for the Care and Use of Laboratory Animals and the Guidelines and Policies for Rodent Experiments provided by the Assessment and Accreditation of Laboratory Animal Care. Human PD-1 KI mice were maintained in a specific pathogen free facility at the animal center of Biocytogen Pharmaceuticals Co., Ltd. (Beijing, China) in individually ventilated cages. Mice were given free access to food and water and maintained on a 12 h light/dark cycle. All mice were euthanized with carbon dioxide (CO_2_) gas using closed chamber in accordance with the guidelines set by the IACUC.

### Cell line

Chinese hamster ovary (CHO) cells (11,619, Gibco, Grand Island, NY, USA) were cultured in DMC7 medium composed of DMEM with high glucose (SH30243.01, HyClone, Captiva) supplemented with 7% FBS (Cat. #, Gibco, Grand Island, NY, USA) and non-essential amino acids (SH30238.01, HyClone, Captiva) and antibiotics-antimycotic solution (SV30079.01, HyClone, Captiva). To generate stable CHO cell lines expressing full-length hPD-1 or mPD-1, each gene construct in the pCMV3 mammalian expression vector (HG10377-CF, MG50124-CF, Sino Biological Inc.) was transfected by Lipofectamine 2000 reagent (11,668,019, Invitrogen,).

The MC38 murine colorectal cancer cell line (ENH204-FP) was purchased from Kerafast, and B16F10 murine melanoma cell line (CRL-6475) was purchased from the ATCC. The Pan02 murine pancreatic cancer cell line was kindly provided by Dr. Chae Gyu Park at Yonsei University College of Medicine (Seoul, Republic of Korea). These cell lines were maintained in Dulbecco’s Minimal Essential Medium (Gibco, Carlsbad, CA) supplemented with 10% FBS (Gibco), 100 U/ml penicillin and 100 μg/ml streptomycin (Gibco) at 37 °C in a humidified atmosphere of 5% CO_2_ and 95% air.

### Antigen

To generate protein antigen of hPD-1, the ECD of hPD-1 gene was fused in frame with soluble FLAG tag, internal ribosomal entry site (IRES), and enhanced green fluorescence protein (EGFP) which was cloned into a mammalian expression vector (6085–1, Clontech). The construct was transfected into CHO cells and treated with G418 (345,812, EMD Millipore) for 1 week. The EGFP-positive CHO cells were enriched by FACS Aria II cell sorter (BD Biosciences), and the FACS-sorted EGFP-high CHO/hPD-1 ECD cells were cloned by limiting dilution. To select CHO/hPD-1 ECD clones, we measured the levels of EGFP expression and soluble hPD-1 ECD secretion by FACSVerse (BD Biosciences) and anti-FLAG western blot, respectively. The soluble hPD-1 ECD protein was purified from the culture supernatant of CHO/hPD-1 ECD cells by an anti-FLAG M1 agarose affinity gel (A4596, Sigma).

To generate cellular antigen of hPD-1, the gene construct in the pCMV3 mammalian expression vector (Sino Biological Inc.) was transfected into BALB/c mouse-originated CT26 cells by Lipofectamine 2000 reagent (11,668,019, Invitrogen).

### Immunization

Mice were used at 8 weeks of age for experiments. Six SHINE MOUSE™ female mice were immunized subcutaneously (s.c.) with 50 μg of purified hPD-1 ECD protein mixed with adjuvant TiterMax® Gold Adjuvant (T2684, Sigma, MO, USA) or intraperitoneally (i.p.) with irradiated CT26 cells (1 × 10^6^ cells per mouse) expressing hPD-1 on the cell surface in turns. Each mouse was immunized 9 times at 3 weeks intervals. Serum samples were collected from the mice 10 days after each immunization. The titer of anti-hPD-1 antibodies in each serum was measured by ELISA. For final boosting, mice were injected intravenously (i.v.) with 10 μg of hPD-1 ECD protein without adjuvant. Three days later, mice were euthanized for production of hybridomas.

### Selection of human/mouse cross-reactive anti-PD-1 antibody and its humanization

Hybridomas were produced from the splenocytes of the immunized mice by fusion with myeloma cells (sp2/0), and hPD-1-specific monoclonal antibody-producing cells were screened by rhPD-1-based ELISA. The final human and mouse cross-reactive anti-PD-1 (clone # 1G1) was selected by cellular PD-1 based ELISA from the supernatants of all the hybridomas. Antibody humanization services were provided by GenScript (New Jersey, NJ), as described in Kurella and Gali [23]. Briefly, for minimizing immunogenicity and excluding effector functions based on its mode of action to better induce proliferation of T effector (Teff) cells, all six complementarity-determining regions (CDR) of the selected 1G1 were grafted into human IgG framework sequences. This was followed by select point mutations to retrieve the original binding affinity and cross-reactivity of the variable region of GNUV201. Finally, human IgG4 (S228P variant) was fused in-frame as the constant region of GNUV201 [24].

### Enzyme-linked immunosorbent assay (ELISA)

For recombinant PD-1-binding ELISA, plates were precoated with 10 ng protein (PD-1 of various species or various immune checkpoint proteins) at 4 °C overnight. After 2 h of blocking with 5% BSA, testing antibodies were incubated at room temperature for 1 h. The bound antibodies were detected by an HRP-conjugated goat-anti-mouse IgG antibody and TMB (3,3',5,5'-tetramethylbenzidine) substrate at 450 nm and 650 nm using a microplate reader.

For cellular PD-1 binding ELISA, PD-1 expressing CHO-S cells were incubated on collagen-coated plates at 4 °C overnight and fixed with paraformaldehyde at room temperature for 15 min. After 2 h of blocking, testing antibodies were incubated at 37 °C for 1 h. The bound antibodies were detected by an HRP-labeled goat-anti-mouse IgG or mouse-anti-human IgG antibody and TMB substrate at 450 nm and 650 nm.

### Surface plasmon resonance (SPR)

SPR analysis was performed at room temperature using a BIAcore 8 K system (Cytiva). For all measurements, an HBS-EP plus buffer (0.1 M HEPES, 1.5 M NaCl, 0.03 M EDTA and 0.5% v/v Surfactant P20 (pH 7.4)) was used as running buffer. For binding at low pH condition, running buffer was adjusted to pH 6.0 with HCl.

For 1:1 (Fab: Antigen) binding assay, the captured anti-PD-1s on protein A chip were incubated with five concentrations (100, 50, 25, 12.5, and 6.25 nM) of hPD-1 (PD-1-H5221, ACRO Biosystems).

For 1:2 (whole IgG: Antigen) binding assay, the CM5 sensor chip (BR-1005–30, Cytiva) was activated with EDC (1-ethyl-3-(3-dimethylaminopropyl)-1-carbodiimide hydrochloride) and NHS (N-hydroxysuccinimide) immediately before use. hPD-1 in 10 mM sodium acetate (pH 5.0) was directly immobilized on the CM5 sensor chip. The chip was deactivated by 1 M ethanolamine HCl. Five concentrations (100, 50, 25, 12.5, and 6.25 nM) of anti-PD-1s were then flowed over the chip surface. For mouse PD-1 binding assay, anti-His antibody (28,995,056, Cytiva) in 10 mM sodium acetate (pH 4.5) was directly immobilized on the CM5 sensor chip. His-tagged mPD-1 (PD1-M5228, ACRO Biosystem) was captured on the CM5 sensor chip and five concentrations (100, 50, 25, 12.5, and 6.25 nM) of anti-PD-1s were then flowed over the chip surface.

### Epitope mapping

Shotgun Mutagenesis epitope mapping services were provided by Integral Molecular (Philadelphia, PA) as described in Davidson and Doranz, 2014 [25]. Briefly, a mutation library of hPD-1 was created by high-throughput, site-directed mutagenesis. Each residue was individually mutated to alanine, with alanine codons mutated to serine. The mutant library was arrayed in 384-well microplates and transiently transfected into HEK293 T cells. Following transfection, cells were incubated with the indicated antibodies at pre-determined concentrations. Bound GNUV201 or control anti-PD-1 were detected using an Alexa Fluor 488-conjugated secondary antibody, and mean cellular fluorescence was determined using Intellicyt iQue flow cytometry platform. Mutated residues were identified as being critical to the GNUV201 epitope if they did not support the reactivity of GNUV201 but did support the reactivity of control anti-PD-1 as a reference. This counter-screening strategy facilitates the exclusion of mutants that are locally misfolded or that have an expression defect.

### Co-crystallization and structure determination of PD-1/GNUV201 Fab complex

ECD of purified recombinant hPD-1 and Fab fragment of GNUV201 were mixed in 1.3:1 molar ratio and incubated for 1 h at 4 °C before being subjected to size exclusion chromatography equilibrated with 20 mM Tris, 150 mM NaCl (pH 7.4). Gel-filtration fractions containing the PD-1/GNUV201 Fab complex were concentrated to 16 mg/ml in 20 mM Tris and 150 mM NaCl (pH 7.4). Crystals of the complex were grown within a week using the hanging-drop vapor diffusion method with a reservoir solution containing 100 mM Tris, 0.05 M lithium sulfate, 45% w/v PEG200 (pH 7.3) at 20 °C. Crystals were cryoprotected by brief immersion in well solution supplemented with 25% glycerol and flash frozen in liquid nitrogen. X-ray diffraction data were collected at 100 K on beamline 5C of the Pohang Light Source (PLS), Republic of Korea. X-ray diffraction data were collected to a resolution of 2.30 Å. The structure was solved by molecular replacement using the CCP4 package. Iterative rounds of refinement were performed using PHENIX with manual inspection using COOT (R/R_free_ = 0.194/0.239) [26–28].

### PD-1/PD-L1 blocking assay

PD-1-expressing CHO-S cells were incubated with serial dilutions of anti-PD-1 at 4 °C for 15 min followed by human Fc (IgG1)-labeled PD-L1 addition. The mixture was incubated at 4 °C for 1 h. Antibodies and PD-L1 were diluted in running buffer (1 × PBS/2% BSA). PE-labeled rat-anti-human IgG1/3 antibody was used to detect the binding of PD-L1 to the cells. The mean fluorescence intensity of cells was measured by a flow cytometer and analyzed by FlowJo (V10.6).

For reporter assay, human PD-L1 aAPC/CHO-K1 cells were incubated in a 96-well plate at 37 °C overnight. Anti-PD-1 and effector cells (Jurkat T cells expressing hPD-1 and a luciferase reporter driven by an NFAT response element (NFAT-RE)) were diluted with assay buffer (RPMI 1640/1% FBS). Anti-PD-1 and effector cells were sequentially added to target cell-containing wells. After 6 h incubation at 37 °C, PD-1/PD-L1 blocking activity was determined by Bio-Glo™ Luciferase Assay kit (G7940, Promega, Southampton, UK)).

### MLR assay and cytokine release with allogenic human PBMC pairs

Frozen peripheral blood mononuclear cells (PBMCs) from normal healthy donors were purchased from Lonza (4W-270, Verviers, Belgium). The CD14^+^ monocytes were isolated by positive selection using CD14 microbeads (130–118-906, Miltenyi Biotec, Auburn, CA, USA) according to the manufacturer’s instructions. The CD14^+^ selected cells were cultured in RPMI 1640 (Gibco) containing 10% FBS (Gibco), 2 mM L-glutamine (Gibco), 1% PS (100 units/ml penicillin and 100 μg/ml streptomycin; Gibco) and supplemented with 50 ng/ml of granulocyte–macrophage colony-stimulating factor (GM-CSF) and 50 ng/ml of interleukin-4 (IL-4; HDC, PeproTech EC Ltd., London, UK) in a humidified 5% CO_2_ incubator. On day 4, monocyte derived dendritic cells (DCs) were stimulated with 1 μg/ml of lipopolysaccharide (LPS; 00–4976-93 Invitrogen, Carlsbad, CA, USA) for 24 h. Frozen PBMCs (Lonza) were cultured in RPMI 1640 (Gibco) containing 10% FBS (Gibco), 2 mM L-glutamine (Gibco), 1% PS (Gibco) and supplemented with T cell TransAct™ (130–128-758, Miltenyi Biotec) for 3 days, and then cells were cultured with fresh medium for 2 days. On day 5, the CD3^+^ T cells were isolated from cultured cells by negative selection using Pan T cell isolation kit (130–096-535, Miltenyi Biotec) according to the manufacturer’s instructions. Isolated CD3^+^ T cells were labeled with CFSE (C34554, Invitrogen) at room temperature for 8 min, washed, and counted before co-culture with mature DCs. The MLR assay was performed by co-culturing 1 × 10^5^ CFSE-labeled T cells with allogeneic monocyte-derived DCs at a ratio of 10:1 (T:DC) in 96 well round-bottom microtiter plates (Corning). T cells and DCs were incubated for 3–5 days in the presence or absence of isotype control or anti-PD-1 (GNUV201, Keytruda^®^, or Opdivo^®^) titrated 1:10 dilutions from 10 μg/ml to 0.1 ng/ml. Cells were harvested on day 5 for FACS analysis of cell proliferation. Culture supernatants were harvested on day 3 or 5 for LEGENDplex™ (741,042, Biolegend Inc., San Diego, CA, US) analysis of IL-2 or IFN-γ secretion, respectively. Comparison was made to responses generated by isotype control treatment.

### In vivo efficacy

MC38 mouse colorectal cells (1 × 10^6^ cells/mouse) were s.c. implanted into the flank of female C57BL/6 mice (Orientbio, Seongnam, Republic of Korea) for validating mPD-1 cross-reactive efficacy of GNUV201 and into the flank of hPD-1 KI female mice (B-hPD-1 plus mice, 110,019, Biocytogen) for direct comparison with approved hPD-1-specific Keytruda^®^ and Opdivo^®^. B16F10 metastatic mouse melanoma cells (1 × 10^6^ cells/mouse) and Pan02 pancreatic cancer cells (3 × 10^6^ cells/mouse) were also s.c. implanted into the flank of female C57BL/6 mice. Mice were randomized at 100 mm^3^ mean tumor volumes and dosed intraperitoneally (i.p.) with 1–10 mg/kg of isotype control or anti-PD-1s twice a week for 2–3 weeks. Tumor size and body weight were monitored twice a week during and after treatment periods. Tumor size was measured in two dimensions using a caliper, and the volume was expressed in mm^3^ using the formula: V = 0.5 × L × W^2^ where L and W are the length and width of the tumor, respectively. Tumor growth inhibition (TGI) was calculated for each group using the following formula: TGI (%) = [1-(T_i_-T_0_)/(V_i_-V_0_)] × 100 (%) (T_i_ and V_i_: the mean tumor volume of the treatment group and the isotype control group, respectively, on a given day; T_0_ and V_0_: the mean tumor volume of the treatment group and the isotype control group, respectively, on day 0). Mouse survival was monitored daily for 56 days. When tumor reached 2,500 mm^3^ in mean volume and/or mice showed signs of poor body condition including respiratory distress, hypoactivity, and failure to respond to stimuli, the mice were sacrificed by CO_2_ inhalation and the date was recorded to calculate survival rate.

### Statistical analysis

Data were expressed as the mean ± standard error (SE). Significance was determined via one‐way ANOVA using PRISM^®^ 5.0 software (GraphPad Software, La Jolla, CA, USA). A two‐tailed Student’s t-test was used to compare data between two groups. Two‐way ANOVA with Bonferroni post-hoc test was used to compare tumor size at multiple time points within groups. Differences in mouse survival rates were determined by a log-rank (Mantel-Cox) test of the Kaplan–Meier survival curves. A value of *P* < 0.05 was regarded as statistically significant.

## Results

### GNUV201 has h/mPD-1 cross-reactivity and differentiated antigen binding properties

**Fig. 1:**
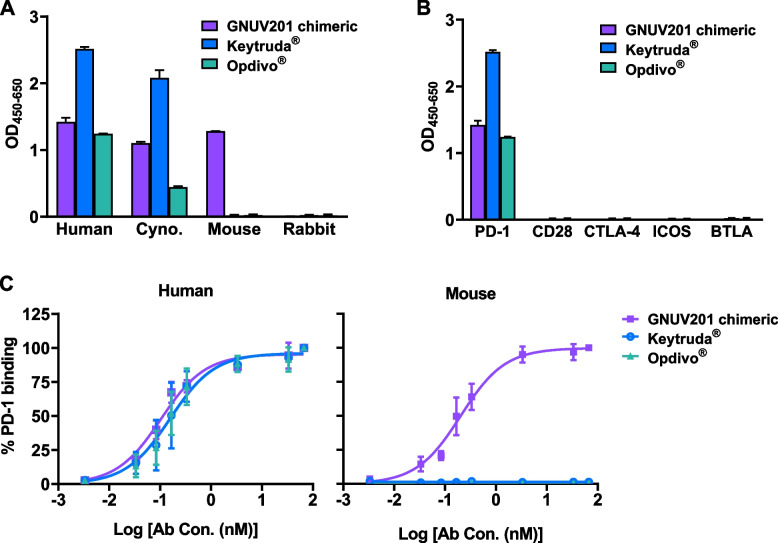
Chimeric GNUV201 has human/monkey/mouse cross-reactivity and selectively binds to hPD-1. (**a** Binding of anti-PD-1s to PD-1 of various species was assessed by ELISA. (**b**) Binding of anti-PD-1s to human PD-1, CD28, CTLA-4, ICOS, or BTLA was assessed by ELISA. (**c**) Binding of anti-PD-1s to cellular h/mPD-1 was assessed by each PD-1 expressing CHO cell-based ELISA. All experiments were performed with at least three independent replicates. Data were fitted to a 4PL curve using GraphPad Prism^®^ software

We immunized SHINE MOUSE™ alternately with hPD-1-expressing CT26 cells and recombinant ECD proteins of hPD-1. SHINE MOUSE™, an enhanced diversity mouse platform with reduced immune tolerance, was used to induce diverse and high immune responses to hPD-1 antigen. After screening hybridoma clones that were generated by fusing activated B lymphocytes isolated from the immunized mice with immortal myeloma cell lines, we successfully identified a h/mPD-1 cross-reactive mouse IgG1 (clone # 1G1). To minimize Fc-mediated effector functions for proper induction of effector T cell (Teff) proliferation, chimeric GNUV201 that contains both the variable region from a mouse 1G1 clone and the constant region from a hinge-stabilized human IgG4 (S228P variant) was prepared. Chimeric GNUV201 equally binds to human, monkey, and mouse PD-1, while Keytruda^®^ and Opdivo^®^ bind only to human and monkey PD-1 (Fig. 1a). Chimeric GNUV201 selectively binds hPD-1 but not to other immunoglobulin superfamily immune checkpoint proteins such as CD28, CTLA-4, ICOS, and BTLA as purified recombinant proteins (Fig. 1b). The unique inter-species cross-reactivity of chimeric GNUV201 for h/mPD-1 was also confirmed by hPD-1- and mPD-1-expressing CHO cell-based ELISAs with comparable EC_50_ values (117 pM and 203 pM for hPD-1 and mPD-1, respectively), while Keytruda^®^ and Opdivo^®^ did not interact with mPD-1 (Fig. 1c, Table 1).

**Table 1:** Affinity of anti-PD-1s to hPD-1 measured by ELISA

		GNUV201 chimeric	Keytruda^®^	Opdivo^®^
hPD-1	EC50 (pM)	117 ± 11	170 ± 25	174 ± 23
	*p*-value	< 0.0001	< 0.0001	< 0.0001
mPD-1	EC50 (pM)	203 ± 19	no binding	no binding
	*p*-value	< 0.0001		

Data were derived from three independent experiments

**Fig. 2:**
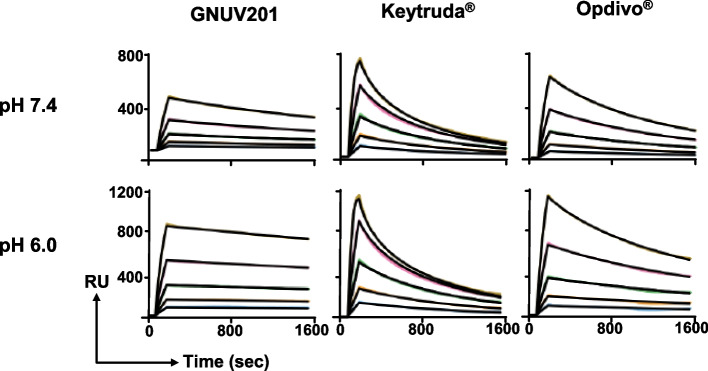
GNUV201 has differentiated antigen binding kinetics. SPR assay characterization of the binding affinity of GNUV201, Keytruda^®^, and Opdivo^®^ to hPD-1 at pH 7.4 and pH 6.0. Interactions of soluble PD-1 with immobilized GNUV201, Keytruda®, and Opdivo^®^ were measured. Realtime SPR sensorgrams were assayed using BIAcore. Y-axis, response unit (RU). X-axis, reaction time course, seconds. A representative graph from four independent experiments is shown

Chimeric GNUV201 was humanized into GNUV201 without significant change in terms of its binding affinity and cross-reactivity using conventional CDR grafting method to minimize immunogenicity. When compared with other approved anti-PD-1s, the binding affinity of GNUV201 to hPD-1 was similar as expected as in the case of GNUV201 chimeric (Fig. 2 and Table 2, K_D_ = 5.86, 7.01, and 7.22 nM for GNUV201, Keytruda^®^, and Opdivo^®^, respectively). Furthermore, GNUV201 dissociates significantly slower from hPD-1 than Keytruda^®^ at pH 7.4 (Table 2, 8.7-fold lower K_off_, *P* < 0.0001), supporting the possibility of better receptor occupancy in vivo.

**Table 2:** Affinity of anti-PD-1s to hPD-1 measured by SPR

		GNUV201	Keytruda^®^	Opdivo^®^	
pH 7.4	*k*_on_ (10^5^/MS)	0.71 ± 0.05	5.02 ± 0.32	1.56 ± 0.10	
	*k*_off_ (10^–3^/s)	0.40 ± 0.01	3.48 ± 0.18	1.12 ± 0.04	
	K_D_ (nM)	5.86 ± 0.47	7.01 ± 0.28	7.22 ± 0.22	
pH 6.0	*k*_on_ (10^5^/MS)	2.04 ± 0.28	10.9 ± 1.09	3.37 ± 0.15	
	*k*_off_ (10^–3^/s)	0.22 ± 0.05	5.25 ± 0.32	1.02 ± 0.08	
	K_D_ (nM)	1.04 ± 0.08	4.99 ± 0.41	3.07 ± 0.34	

Data are presented as mean ± standard error of four independent experiments

Surprisingly, while immobilized GNUV201 (K_D_ = 5.86 nM) interacting with hPD-1 in the soluble phase showed comparable binding affinity with marketed Keytruda^®^ (K_D_ = 7.01 nM) at neutral pH 7.4, the affinity became significantly stronger at TME-mimicking pH (pH 6.0) by about fivefold (GNUV201: 1.04 nM vs. Keytruda^®^: 4.99 nM, Table 1, *P* < 0.0001). The difference was mainly due to the slower dissociation rate of GNUV201 at pH 6.0, unlike other anti-PD-1s which do not show meaningful change between the two different pH levels.

The low pH selectivity of GNUV201 was well maintained when GNUV201 was in the soluble phase, which is more relevant to the actual dosing condition to demonstrate the antibody's avidity. (Fig. S1). In this condition that simulates divalent anti-PD-1 and PD-1 interactions, GNUV201 showed even stronger binding affinity at pH 6.0 compared to Keytruda^®^ and Opdivo^®^ (about 7 to tenfold, K_D_ = 0.10 nM, 0.67 nM, *P* = 0.0158), and 1.03 nM, *P* = 0.0004) for GNUV201, Keytruda^®^, and Opdivo^®^ respectively), where only GNUV201 exhibited slower dissociation rate at pH 6.0 compared to pH 7.4 (Fig. S1A). Both Keytruda^®^ and Opdivo^®^ showed faster dissociation rates at pH 6.0 than at pH 7.4, implying that GNUV201 achieves pH-dependent affinity enhancement. The enhancement of affinity at low pH due to the lower dissociation of GNUV201 was also observed for mPD-1 (Fig. S1B). This property of GNUV201 could result in TME-selective PD-1 binding for better occupancy on PD-1 in tumors compared to its marketed antibodies.

### GNUV201/hPD-1 epitope mapping and co-complex results show its unique epitope and support its cross-reactivity

**Fig. 3:**
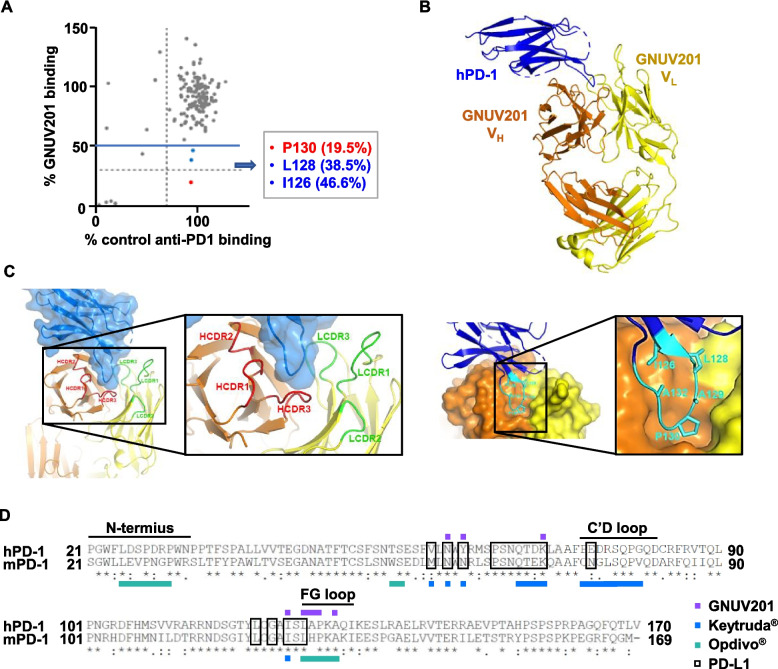
GNUV201/hPD-1 epitope mapping and co-crystallization results show its unique epitope and support its cross-reactivity. **a** Epitope mapping by alanine-scanning results of PD-1 in terms of GNUV201 bind-ability. The comparative binding of non-functional control anti-PD-1 and GNUV201 to PD-1 was graphed (Cutoff: GNUV201 binding < 50%). **b** Co-crystal structure (2.30 Å) of the Fab of GNUV201 (heavy chain: orange, light chain: yellow) and PD-1 (blue). **c** Major interaction of GNUV201 (heavy chain: orange, light chain: yellow) with the FG loop (cyan) of PD-1(blue). **d** Sequence alignment of human and mouse PD-1. The PD-1 site that binds to PD-L1 is indicated by a black box. Epitopes of anti-PD-1s are marked with different colors (GNUV201: purple dot, Keytruda^®^: blue dot, Opdivo^®^: cyan dot)

The cross-reactivity of GNUV201 and its slow dissociation rate raise interest on the binding mechanism and epitopes of GNUV201. To understand the binding characteristics of GNUV201 at the structural level, we performed alanine scanning on the ECD of hPD-1 (F24-V170) to determine the contribution of specific residues to the binding of GNUV201 to its antigen, hPD-1. The alanine residue on hPD-1 was mutated to serine, while each of the other residues were mutated to alanine. The comparative binding results between PD-1 (WT vs. Ala mutants)-expressing cells and anti-PD-1 (non-blocking control antibody vs. GNUV201) were analyzed by flow cytometry. The most functionally important mutations on hPD-1 that significantly reduced GNUV201-binding (< 50%) were three mutants (P130A (19.5% of WT PD-1 control), L128A (38.5%), and I126A (46.6%)) (Fig. 3a). To identify the exact binding mode of action, we co-crystallized the complex of GNUV201 Fab fragment with hPD-1 and solved the complex structure at a resolution of 2.30 Å (Fig. 3b). The complex structure revealed that GNUV201 utilized five out of six CDRs to interact with PD-1 (Fig. 3c). The three CDR loops in the variable region of the heavy chain (V_H_) are mainly involved in the interaction with PD-1. More specifically, all the residues involved in hydrogen bonding for the interactions between GNUV201 and hPD-1 are only on the three CDR loops of the heavy chain (Fig. S2a). On the other hand, two CDR loops in the variable region of the light chain (V_L_) only contribute to hydrophobic interactions in the complex of GNUV201 and hPD-1 (Fig. S2b). These results imply that the overall interaction between GNUV201 and hPD-1 is primarily led by the heavy chain.

The epitope recognized by GNUV201 is mainly located on the FG loop region of hPD-1 (Fig. 3C), which is well conserved in both hPD-1 and mPD-1, supporting GNUV201’s inter-species cross-reactivity (Fig. 3d); this position is distinct from those of Keytruda^®^ (C’D loop) and Opdivo^®^ (N-term). All three important residues (P130, L128, I126) identified from alanine scanning mutagenesis are also located in the FG loop of hPD-1, and P130, the most critical residue for binding of GNUV201 to hPD-1, is located at the tip of the FG loop (Fig. 3c). The amino acids comprising the GNUV201 epitope identified from both the crystal structure and alanine scanning mutagenesis are well conserved between human and mouse when whole amino acid sequences of the ECD of hPD-1 and mPD-1 are aligned and compared (Fig. 3d). Among them, 6/8 (75%) are identical in human and mouse, again supporting inter-species cross-reactivity of GNUV201.

The binding residues that differ between human and mouse are the 68th and 129th amino acids of PD-1. Y68 of hPD-1 is hydrogen-bonded with the backbone O atom of heavy chain S55, but N68 of mPD-1 is short in length, so the binding ability may be lowered due to its inability to generate a hydrogen bond. On the other hand, A129 of hPD-1 has a weak hydrophobic interaction with light chain Y37, but H129 of mPD-1 seems to be able to form a new hydrogen bond with Y37 (Fig. S2c). As no other structural conflicts were found, GNUV201 appears to have h/m cross-reactivity.

###  GNUV201 enhanced T cell proliferation and cytokine secretion via complete blocking of PD-1/PD-L1 interaction

From the structure of the binding complex, it is possible to explain the mechanism of action of GNUV201 as a blocking antibody. GNUV201 (surface model) bound to PD-1 (blue) collides with PD-L1 (red) binding to PD-1 (Fig. S2d). Since the binding ability of GNUV201 to PD-1 is much stronger than that of PD-L1 (K_D_ = 4.1 μM, [29]), it can block PD-1/PD-L1 interactions completely (Fig. S2d).

**Fig. 4:**
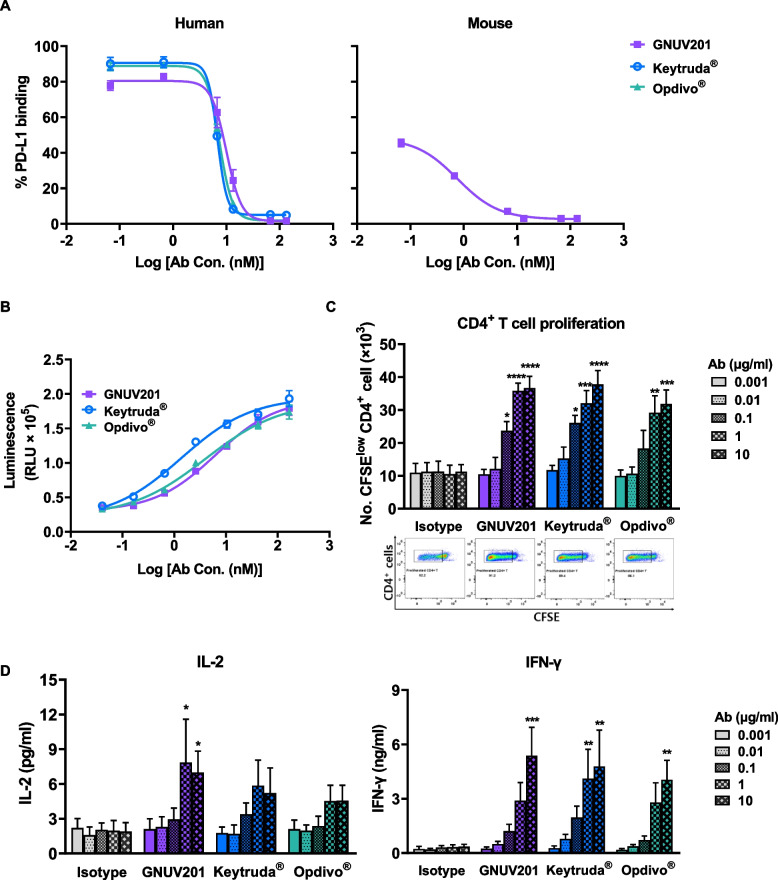
GNU201 blocks PD-1/PD-L1 interactions and potently enhances proliferation and activated cytokine secretion of Teff cells. **a**) The ability of anti-PD-1s to block h/mPD-1/PD-L1 interactions was assessed by flow cytometry. **b** The ability of anti-PD-1s to block hPD-1/PD-L1 interactions was assessed by reporter assay. Data were fitted to a 4PL curve using GraphPad Prism^®^ software. **c** and **d** The ability of anti-PD-1s to enhance T cell proliferation and cytokine secretion was assessed using allogeneic MLR assay. Allogeneic MLR assays were performed using monocyte-derived DCs and T cells from 4 donors at a ratio of 1:10 (DC:T cell). CFSE-labeled CD4^+^ T cells and DCs prepared from different donors of hPBMCs were incubated for 5 days in the presence of respective concentrations of each antibody. Proliferation of CD4^+^ T cells (CFSE-dilutions) was analyzed by flow cytometry, and representative CFSE-dilutions (5 days, 10 ug/ml) are plotted below the graph. Culture supernatants were harvested on day 3 for LEGENDplex™ analysis of IL-2 and IFN-γ secretion. Data represent mean ± standard error of four independent experiments. * *P* < 0.05; ** *P* < 0.01; *** *P* < 0.001; **** *P* < 0.0001 compared with the isotype group (two-way ANOVA with Tukey’s multiple comparisons test)

The blocking of PD-1/PD-L1 interaction by GNUV201 was evaluated by flow cytometry and reporter assay. GNUV201 can completely block hPD-1 binding to hPD-L1 with IC_50_ of 9.9 nM (Fig. 4a left panel, flow cytometry) and 6.3 nM (Fig. 4b, reporter assay), comparable to Keytruda^®^ and Opdivo^®^. GNUV201 also blocks mPD-1 binding to mPD-L1 with IC_50_ of 6.7 nM (Fig. 4a right panel, flow cytometry). GNUV201 equally binds to h/mPD-1 and blocks h/mPD-1 binding to PD-L1.

Blocking the interaction of PD-1 with PD-L1 results in the restoration of exhausted T cells, which causes T cell proliferation, cytokine secretion, cytotoxicity, and normalization of anti-tumor response, the rationale behind immune checkpoint blockades [30]. Therefore, the ability of GNUV201 to reinvigorate T cell function was measured using the allogeneic human MLR assay. GNUV201 significantly increased T cell proliferation and cytokine (IL-2 and IFN-γ) release from T cells in a dose-dependent manner similar to Keytruda^®^ (Fig. 4c and d). In summary, GNUV201 nicely promotes T cell proliferation and cytokine secretion in human system in vitro.

### GNUV201 significantly inhibited tumor growth in several immunocompetent mouse syngeneic and hPD-1 KI mice models

**Fig. 5:**
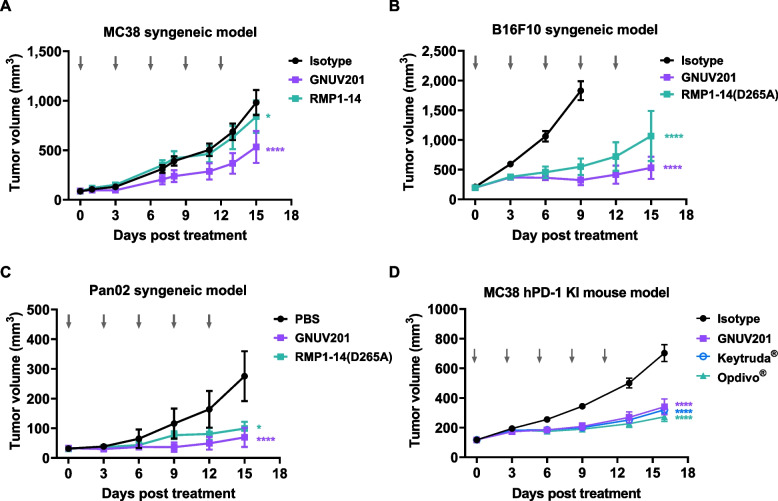
GNUV201 inhibits in vivo tumor growth in murine colorectal, melanoma, and pancreatic cancer models, C57BL/6 mice (n = 5—10/group) were s.c. injected with 1 × 10^6^ MC38 tumor cells (**a**), 1 × 10^6^ B16F10 tumor cells (**b**), and 3 × 10^6^ Pan02 tumor cells (**c**). **d** hPD-1 KI mice (n = 13/group) were s.c. injected with 5 × 10^5^ MC38 tumor cells. Mice were i.p. treated every 3 days with GNUV201, RMP1-14, RMP1-14(D265A), Keytruda^®^, Opdivo^®^, or isotype with a dosing schedule indicated by arrows. Tumor volume was assessed every 3 days following treatment, and tumor volume is shown as mean ± standard error. * *P* < 0.05; **** *P* < 0.0001 compared with the isotype group (two-way ANOVA with Bonferroni post-hoc test)

The in vivo anti-tumor efficacy of GNUV201 was evaluated using MC38 colorectal cancer, B16F10 melanoma, and Pan02 pancreatic cancer model in C57BL/6 mouse (Fig. 5a-c) and MC38 colorectal cancer model in hPD-1 KI mice (Fig. 5d). Tumor growth inhibition (TGI) was calculated on day 15 post-treatment. In the MC38 syngeneic model, GNUV201 showed inhibition of tumor growth in a dose-dependent manner (Fig. S3a and Fig. 5a). Compared with the isotype control, GNUV201 exhibited significant TGI of 24%, 59%, and 73% at 1, 3, and 10 mg/kg, respectively. Overall survival improvement was statistically significant (*P* < 0.005), and about 20% of the mice treated with GNUV201 (10 mg/kg) survived without any signs of tumor growth (Fig. S3b). Therefore, all subsequent experiments were tested with the 10 mg/kg dose of anti-PD-1s. GNUV201 markedly reduced tumor growth enough to induce regression of tumor during the administration period in the B16F10 and Pan02 syngeneic model (Fig. 5b; 89% and 75% TGI, respectively). In order to compare anti-tumor efficacy with marketed antibodies, Keytruda^®^ and Opdivo^®^ which recognize only hPD-1, we used hPD-1 KI mice. This mouse model has the advantage of expressing the hPD-1 protein in the context of a fully functional immune system, which enables in vivo efficacy testing of immune checkpoint inhibitors. In the hPD-1 KI mouse, GNUV201 significantly inhibited MC38 tumor growth (55.9% TGI, *P* < 0.0001); its anti-tumor effect is comparable to that of Keytruda^®^ and Opdivo^®^ (Keytruda^®^: 57.4% TGI, *P* < 0.0001, Opdivo^®^: 63.9% TGI, *P* < 0.0001, GNUV201 vs Keytruda^®^ and Opdivo^®^*, P* > 0.4) (Fig. 5d). No abnormal body weight changes or signs of toxicity were observed throughout the study. Hence, GNUV201 showed strong and consistent in vivo efficacy in all of the MC38, B16F10, and Pan02 syngeneic models, and in the MC38 hPD-1 KI model (Fig. 5).

## Discussion

Therapeutic antibodies are developed for human use, but the efficacy and safety in the preclinical phase need to be evaluated in relevant animal models. Several alternative methods/systems have been devised to test the efficacy of therapeutic antibodies which only recognize human antigens. For example, surrogate antibody binding to a mouse target can be used. However, since the surrogate antibody is not guaranteed to be identical to the therapeutic antibody, the complexities and challenges for translation of preclinical safety and efficacy results to the clinic are undoubtedly aggravated when surrogate approaches are employed in both preclinical and clinical phase. Secondly, the therapeutic antibody can be evaluated in transgenic mice expressing human targets. These mice, however, are not always available at the time when the therapeutic antibody is ready to be evaluated [15]. Therefore, the most accurate and efficient way to validate antibody efficacy and safety is to directly test human/mouse cross-reactive antibodies in mouse models.

We immunized SHINE MOUSE™ with hPD-1 antigen, screened a large number of mouse hybridoma clones, and successfully identified a functional human/mouse cross-reactive antibody, GNUV201. GNUV201 shows equal PD-1 binding affinity and blocking activity of PD-1/PD-L1 interaction in both human and mouse (Fig. 1, Fig. 4a). As expected from the antigen–antibody structure complex, GNUV201 binds to a unique ‘FG loop’ on hPD-1, different from the ‘C’D loop’ and ‘N-term’ of Keytruda^®^ and Opdivo^®^, respectively (Fig. 3). Interestingly, GNUV201 exhibits slow dissociation from the antigen and improved binding affinity at a TME-mimicking low pH, unlike Keytruda^®^ and Opdivo^®^ (Fig. 2, Fig. S1 Table 1). In human mixed lymphocyte reaction (MLR) assay, GNUV201 showed significantly improved T cell proliferation and IFN-γ secretion. GNUV201 shows strong tumor growth inhibition efficacy in mouse models of colorectal cancer (MC38), melanoma (B16F10), and pancreatic cancer (Pan02). It also demonstrates a comparable level of anti-cancer efficacy as Keytruda^®^ and Opdivo^®^ in the MC38 hPD-1 knock-in (KI) mouse model (Fig. 5).

In this study, we showed the properties and efficacy of GNUV201 as a therapeutic antibody with cross-reactivity to both human and mouse. First, the cross-reactivity of GNUV201 is confirmed by the comparison of epitopes identified from the co-crystal structure analysis. The sequence homologies between human and mice epitopes of GNUV201 (75%, 6/8) is much higher than the marketed anti-PD-1 s, Keytruda^®^ (50%, 8/16) and Opdivo^®^ (57%, 8/14), supporting the human/mouse cross-reactivity of GNUV201. In addition, the fact that the FG loop through which GNUV201 binds to PD-1 is known to be very conserved across species [31] and that the FG loop is a relatively narrow epitope compared to those of Keytruda^®^ and Opdivo^®^, which minimizes collisions with different residues of PD-1 in other species, could also support the h/m cross-reactivity of GNUV201.

The CS1003 antibody developed by CStone Pharmaceuticals also has been claimed to have h/m cross-reactivity [32, 33]; however, CS1003 inhibits human and mouse PD-1/PD-L1 interactions with IC_50_ values with a difference of more than 10x[33]. In contrast, GNUV201’s equivalent antigen binding and blocking of PD-1/PD-L1 interaction (equivalent single-digit nM of IC_50_) in human and mouse enable preclinical studies with potentially more accurate validation of efficacy and safety. This in turn would be more precisely translated into efficacy and safety in humans for clinical development.

Co-crystal structures of hPD-1, hPD-L1, and anti-PD-1 antibodies reveal how hPD-1 interacts with its counterparts PD-L1 or anti-PD-1s (Fig. S2e, GNUV201, Keytruda^®^, and Opdivo^®^). Although the epitope area (658 Å^2^) of GNUV201 is smaller than those of Keytruda^®^ and Opdivo^®^, GNUV201 shows better binding kinetics to hPD-1 due to slower dissociation compared to Keytruda^®^ and Opdivo^®^. The binding characteristics of GNUV201 can be interpreted through antibody/antigen complex structure, in which the protruding epitope loop (FG-loop) of hPD-1 fits deeply into GNUV201’s binding pocket formed by the CDRs (Fig. 3c). Through this binding, GNUV201 would not be easily released from its target, PD-1. This interpretation additionally suggests that the most elongated FG loop region on PD-1 may be the most efficient epitope for PD-1 targeting antibodies.

Interactions between antibody and antigen are generally characterized by their affinity and specificity and affected by environmental features, such as pH [34]. Since high affinity to their target is a prerequisite for monoclonal antibodies to achieve therapeutic benefits, it is very important to confirm the affinity of antibodies in TME-mimicking low pH. In general, histidine is the most pH-sensitive residue for binding. A histidine residue on position 52 (H52) of GNUV201 was identified to be hydrogen bonded with PD-1 within the binding interface (Fig. S2a); this is not found in Keytruda^®^ or Opdivo^®^. Histidine is the only amino acid which has a logarithmic acid dissociation constant (p*K*_a_) around 6.0 and can be sensitively protonated and become positively charged or deprotonated (no charge) around physiological pH (~ pH 7.4) to tumor micro-environmental low pH (~ pH 6.0) [35]. This phenomenon suggests that the binding force of GNUV201 to hPD-1 may vary depending on pH. The presence of a histidine residue on GNUV201’s paratope mapped in the structural complex provided evidence that the antigen binding affinity may vary depending on pH, and this has been demonstrated experimentally. Enhanced antigen binding affinity of GNUV201 at low pH suggest the possibility that GNUV201 retains more favorable binding characteristics at low pH of the TME compared to other anti-PD-1 antibodies, which could lead to improved anti-cancer efficacy in terms of longer duration of efficacy or requiring less amount of dosing (Fig. S1).

GNUV201 has an anti-cancer effect equivalent to Keytruda^®^ and Opdivo^®^ in MC38/hPD-1 KI mice and shows a strong tumor regression effect in B16F10 and Pan02 (Fig. 5). Surprisingly, GNUV201 showed complete tumor growth inhibitory efficacy in B16F10 melanoma cells, which develop acidic environments via proton secretion [36] and typically show resistance to PD-1 therapy [37, 38]. This phenomenon suggests the possibility that GNUV201 can have differentiated anti-cancer efficacy even in TME with a harsh environment such as low pH.

The translation of biotherapeutic drug candidates into a usable drug product requires stability that can withstand various stresses during manufacturing, shipping, and storage while retaining acceptable pharmacology, safety, immunogenicity, and toxicity profiles. Currently, several tests for physicochemical and biophysical properties of GNUV201 are underway to confirm whether GNUV201 can be translated into a drug product.

Based on previous studies that have shown that anti-PD-1 antibodies with 'effector-less' Fc produce improved anti-cancer effect, we are engineering Fc variants for further evaluations [39, 40].

## Conclusions

We report the novel antigen-binding characteristics of GNUV201, identified by both SPR analysis and structural biology, and anti-tumor efficacy tests of GNUV201 in tumor-bearing syngeneic mouse models. Distinct from current FDA-approved anti-PD-1s, GNUV201 shows h/m cross-reactivity, improved hPD-1 binding kinetics, especially in low pH, and strong tumor regression efficacy in the B16F10 mouse syngeneic melanoma model. All these findings facilitate our understanding of the potential of GNUV201 as an anti-cancer antibody with novel properties and provide a novel targetable region for the development of anti-PD-1 antibodies for tumor immunotherapy.

### Supplementary Information

**Additional file 1**

## Data Availability

The datasets in this study are available from the corresponding author upon reasonable request.
