# Supplementary material for: GNUV201, a novel human/mouse cross-reactive and low pH-selective anti-PD-1 monoclonal antibody for cancer immunotherapy
Source: BMC Immunol. 2024 May 11;25:29. doi: 10.1186/s12865-024-00609-z (PMC11088064; doi:10.1186/s12865-024-00609-z)
Supplement: Supplementary file 1 — Additional file 1 [file 12865_2024_609_MOESM1_ESM.pdf]

GNUV201, a novel human/mouse cross-reactive and low pH-selective anti-PD-1 monoclonal antibody for cancer immunotherapy

Hae-Mi Kim[a], Kyoung-Jin Kim[a], Kwanghyun Lee[a], Myeong Jin Yoon[a], Jenny Choih[a,b], Tae-Joon Hong[a], Eun Ji Cho[a], Hak-Jun Jung[a], Jayoung Kim[a], Ji Soo Park[c,d], Hye Young Na[c,e], Yong-Seok Heo[f], Chae Gyu Park[a,c], Heungrok Park[a], Sungho Han[a,b], and Donggoo Bae[a,*]

[a]Genuv Inc., 5th Fl. NIA Building, Cheonggyecheon-Ro 14, Jung-gu, Seoul, Republic of Korea; [b]Genuv US Subsidiary, CIC, 1 Broadway, Cambridge, MA, USA; [c]Laboratory of Immunology, Severance Biomedical Science Institute, Yonsei University College of Medicine, Seoul, Republic of Korea; [d]Brain Korea 21 PLUS/FOUR Project for Medical Science, Yonsei University College of Medicine, Seoul, Republic of Korea; [e]Department of Neurology, Severance Hospital, Yonsei University College of Medicine, Seoul, Republic of Korea; [f]Department of Chemistry, Konkuk University, 120 Neungdong-ro, Gwangjin-gu, Seoul, 05029, Republic of Korea; Donggoo Bae and Sungho Han are co-corresponding authors. *To whom correspondence should be addressed. Email: Justin@genuv.com

Supplementary information

A

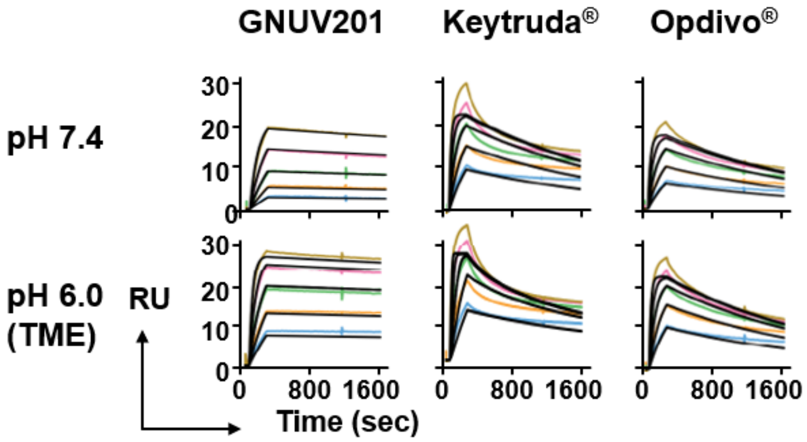

		GNUV201	Keytruda®	Opdivo®
pH 7.4	k_{on} (10^5/MS)	1.49± 0.15	6.74 ± 0.81	5.05 ± 0.54
	k_{off} (10^{-3}/s)	0.06 ± 0.01	0.45 ± 0.06	0.47 ± 0.04
	K_D (nM)	0.41 ± 0.15	0.72 ± 0.17	1.00 ± 0.2
pH 6.0	k_{on} (10^5/MS)	3.15 ± 0.19	23.3 ± 3.09	5.79 ± 0.38
	k_{off} (10^{-3}/s)	0.03 ± 0.01	1.65 ± 0.46	0.59 ± 0.03
	K_D (nM)	0.10 ± 0.02	0.67 ± 0.09	1.03 ± 0.11

B

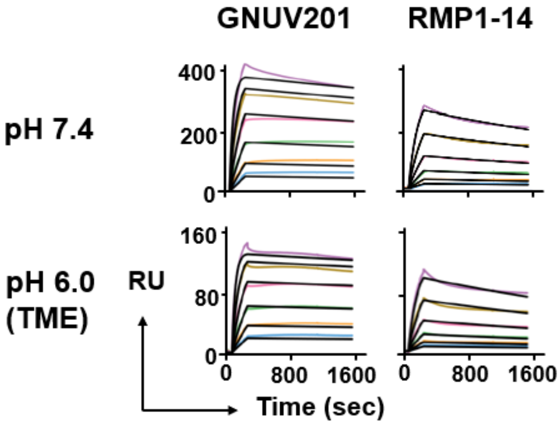

		GNUV201	RMP1-14
pH 7.4	k_{on} (10^5/MS)	2.50 ± 0.02	1.21 ± 0.29
	off (10^{-3}/s)	0.09 ± 0.02	0.28 ± 0.04
	K_D (nM)	0.38 ± 0.09	2.43 ± 0.40
pH 6.0	k_{on} (10^5/MS)	2.82 ± 0.03	0.89 ± 0.03
	off (10^{-3}/s)	0.05 ± 0.01	0.25 ± 0.01
	K_D (nM)	0.18 ± 0.02	2.88 ± 0.24

Supplement Figure 1. GNUV201 has differentiated antigen binding kinetics.

(a) SPR assay characterization of the binding affinity of GNUV201, Keytruda®, and Opdivo® to hPD-1 at pH 7.4 and pH 6.0. Interactions of soluble GNUV201, Keytruda®, and Opdivo® with immobilized PD-1 were measured. (b) SPR assay characterization of the binding affinity of GNUV201 and RMP1-14 to mPD-1 at pH 7.4 and pH 6.0. Interactions of soluble GNUV201 and RMP1-14 with captured PD-1 were measured. Realtime SPR sensorgrams were assayed using BIAcore. Y-axis, response unit (RU). X-axis, reaction time course, seconds. A representative graph from four independent experiments is shown.

A

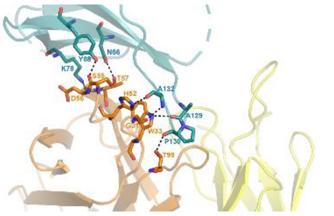

Heavy chain	Light chain	PD-1	Distance (Å)
G31 (O)		A132 (N)	3.01
W33 (NE1)		A129 (O)	2.78
H52 (NE2)		A132 (O)	2.83
T99 (OZ1)		P130 (O)	2.69
S55 (O)		Y68 (OH)	2.51
D56 (O)		K78 (NZ)	2.98
T57 (OG1)		N66 (OD1)	2.64

B

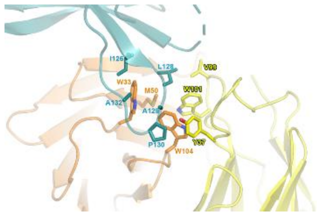

Heavy chain	Light chain	PD-1
W33		I126
W33		L128
W33		A132
	Y37	A129
	Y37	P130
M50		L128
	V99	L128
	W101	L128
W104		L128
W104		P130

C

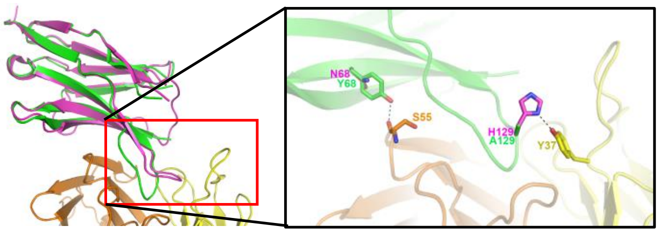

D

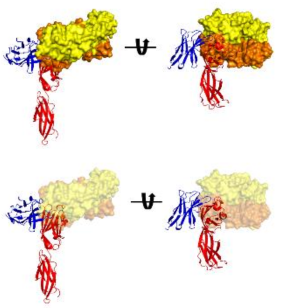

E

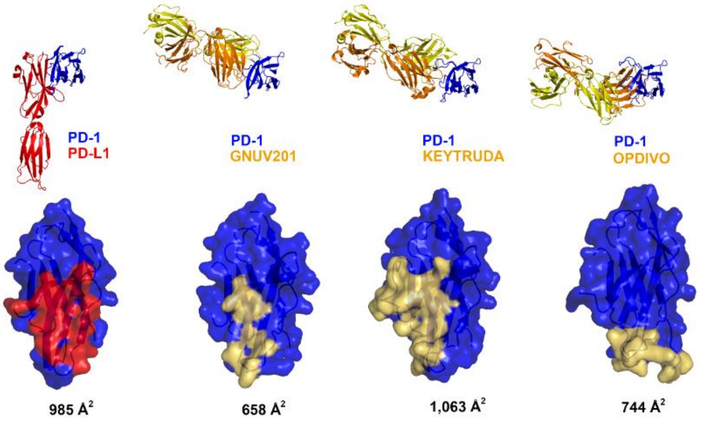

Supplement Figure 2. Detailed interactions between GNUV201 and PD-1.

(a) Hydrogen bonds in the complex of GNUV201 (heavy chain: orange, light chain: yellow) and PD-1 (blue). (b) Hydrophobic interactions in the complex of GNUV201 (heavy chain: orange, light chain: yellow) and PD-1 (blue). (c) Structural basis of human-mouse cross reactivity of GNUV201. (d) Mechanism of action of GNUV201. (e) PD-1 binding comparison of GNUV201, Keytruda®, and Opdivo®.

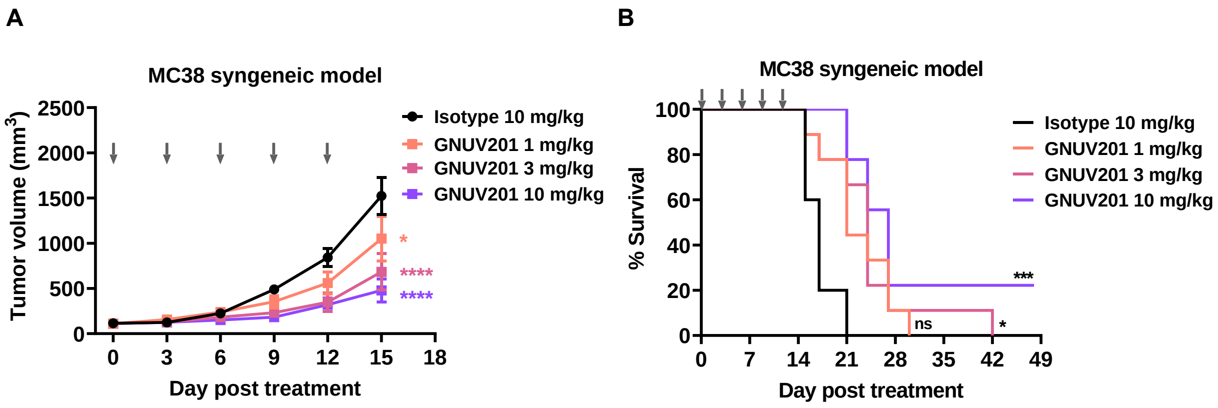

Supplement Figure 3. Treatment of GNUV201 increases tumor growth inhibition and overall survival in a dose-dependent manner.

C57BL/6 mice (n = 5 - 9/group) were s.c. injected with 1 × 10^6 MC38 tumor cells. Mice were i.p. treated every 3 days with GNUV201 (indicated dose) with a dosing schedule indicated by arrows. Tumor volume was assessed every 3 days following treatment, and tumor volume is shown as mean ± standard error (a). * $P < 0.05$; **** $P < 0.0001$ compared with the isotype group (two-way ANOVA with Bonferroni post-hoc test). (b) Survival rate was recorded as the percentage of surviving mice on a given day. * $P < 0.05$; ** $P < 0.005$ compared with the isotype group (Log-rank (Mantel-Cox) test).
